# 3D-Printed Replica and Porcine Explants for Pre-Clinical Optimization of Endoscopic Tumor Treatment by Magnetic Targeting

**DOI:** 10.3390/cancers13215496

**Published:** 2021-11-01

**Authors:** Anjali A. Roeth, Ian Garretson, Maja Beltz, Till Herbold, Maximilian Schulze-Hagen, Sebastian Quaisser, Alex Georgens, Dirk Reith, Ioana Slabu, Christian D. Klink, Ulf P. Neumann, Barbara S. Linke

**Affiliations:** 1 Department of General, Visceral and Transplant Surgery, RWTH Aachen University Hospital, 52074Aachen, Germany; therbold@ukaachen.de (T.H.); christian.klink@diakonissen.de (C.D.K.); uneumann@ukaachen.de (U.P.N.); 2 Department of Surgery, Maastricht University Medical Center, 6229 HX Maastricht, The Netherlands; 3 Department of Mechanical and Aerospace Engineering, University of California Davis, Davis, CA 95616, USA; icgarretson@ucdavis.edu (I.G.); maja.beltz@web.de (M.B.); sequaisser@t-online.de (S.Q.); atgeorgens@ucdavis.edu (A.G.); bslinke@ucdavis.edu (B.S.L.); 4 Department of Electrical and Mechanical Engineering, Bonn-Rhein-Sieg University of Applied Sciences, 53757 Sankt Augustin, Germany; dirk.reith@h-brs.de; 5 Department of Diagnostic and Interventional Radiology, RWTH Aachen University Hospital, 52074 Aachen, Germany; mschulze@ukaachen.de; 6 Institute of Applied Medical Engineering, Helmholtz-Institute Aachen, RWTH Aachen University, 52062 Aachen, Germany; slabu@ame.rwth-aachen.de

**Keywords:** 3D printing, pancreatic cancer, models, replica, magnetic nanoparticles, magnetic hyperthermia, endoscopy, digital manufacturing

## Abstract

**Simple Summary:**

Animal models are often needed in cancer research but some research questions may be answered with other models, e.g., 3D replicas of patient-specific data, as these mirror the anatomy in more detail. We, therefore, developed a simple eight-step process to fabricate a 3D replica from computer tomography (CT) data using solely open access software and described the method in detail. For evaluation, we performed experiments regarding endoscopic tumor treatment with magnetic nanoparticles by magnetic hyperthermia and local drug release. For this, the magnetic nanoparticles need to be accumulated at the tumor site via a magnetic field trap. Using the developed eight-step process, we printed a replica of a locally advanced pancreatic cancer and used it to find the best position for the magnetic field trap. In addition, we described a method to hold these magnetic field traps stably in place. The results are highly important for the development of endoscopic tumor treatment with magnetic nanoparticles as the handling and the stable positioning of the magnetic field trap at the stomach wall in close proximity to the pancreatic tumor could be defined and practiced. Finally, the detailed description of the workflow and use of open access software allows for a wide range of possible uses.

**Abstract:**

Background: Animal models have limitations in cancer research, especially regarding anatomy-specific questions. An example is the exact endoscopic placement of magnetic field traps for the targeting of therapeutic nanoparticles. Three-dimensional-printed human replicas may be used to overcome these pitfalls. Methods: We developed a transparent method to fabricate a patient-specific replica, allowing for a broad scope of application. As an example, we then additively manufactured the relevant organs of a patient with locally advanced pancreatic ductal adenocarcinoma. We performed experimental design investigations for a magnetic field trap and explored the best fixation methods on an explanted porcine stomach wall. Results: We describe in detail the eight-step development of a 3D replica from CT data. To guide further users in their decisions, a morphologic box was created. Endoscopies were performed on the replica and the resulting magnetic field was investigated. The best fixation method to hold the magnetic field traps stably in place was the fixation of loops at the stomach wall with endoscopic single-use clips. Conclusions: Using only open access software, the developed method may be used for a variety of cancer-related research questions. A detailed description of the workflow allows one to produce a 3D replica for research or training purposes at low costs.

## 1. Introduction

The design of an experiment is crucial for its outcome and the corresponding conclusion. In cancer research, animal experiments are often necessary to answer questions regarding living subjects, especially blood flow, pharmacological distribution, etc. Still, not all questions can be answered by animal models, as there are fundamental differences in human and animal anatomy. For example, the liver of rodents is lobed as opposed to the segmented human liver. In addition, there are differences in the portal venous system as well. Hence, for anatomy-specific research questions such as that related to positioning the magnetic field traps for the magnetic targeting of nanoparticles for tumor treatment [1], new models are needed. In accordance with the “3R” principle of reducing, refining and replacing animal experiments as far as possible [2], new models and possibilities have to be taken into account in preclinical evaluations. The rapid advancements of additive manufacturing, also termed 3D printing, and its growing accessibility offer exciting possibilities to fabricate anatomically correct prototypes [3,4,5]. With an example application for tumor treatment by magnetic targeting, we demonstrate a successful experimental design with additive manufacturing and a reduction in animal experiments.

Additive manufacturing processes can be categorized in several ways, such as the feedstock material (polymers, metal, etc.), the feedstock phase and shape (solid-based, liquid-based, powder-based), or the physical working principle (melting, sintering, polymerization, etc.) [6]. The most popular types of additive manufacturing are material extrusion (or fused deposition modeling), vat photopolymerization (or stereolithography) and powder bed processes such as binder jetting, material jetting (polyjet), selective laser sintering or melting, and electron beam melting [6]. The advantages of 3D printing compared with conventional manufacturing include reduced material waste and energy consumption, shortened time-to-market, just-in-time production, and the production of structures not otherwise possible to make [7]. Additive manufacturing promises high degrees of freedom in design as well as customization for individual patients [8,9].

A growing number of applications of additive manufacturing for cancer research and surgery have been described, especially the challenges for 3D printing in cancer applications [10,11]. For example, George et al. successfully fabricated medical instruments (a surgical set including hemostats, needle driver, scalpel handle, retractors and forceps) with selective laser sintering (SLS) [12]. Hong et al. produced a CT-based 3D thyroid cancer phantom using additive manufacturing technology as a physical model, which was used to successfully communicate with patients [3]. From the CT scans, the phantom was produced with the medical image processing software (Mimics and 3-matics; Materialise, Leuven, Belgium), which is discussed by Hong et al. [4]. The models of different vessels were printed in color and the inner part of the thyroid gland where the tumor was located was printed in transparent plastic. With these replicas, they proved that patients could better understand their individual conditions. Similarly, Santiago et al. were able to prove that 3D breast models were a good tool to decrease the decisional conflict in patients with breast cancer [13]. Kong et al. developed several 3D-printed replicas of hepatic segments from CT scans as a teaching aid for young surgeons [5]. A model of hepatic segments without parenchyma and a model of hepatic vessels with segmental partition were printed in color with a powder bed process using 3D systems. For a model of hepatic segments with transparent parenchyma, a mold for the parenchyma was additively manufactured and then filled with transparent jelly wax. In another study, Chen et al. divided patients undergoing right hemicolon cancer surgery into three groups (3D-printing, 3D-image and control) and were able to demonstrate that 3D-printing could reduce the length of surgery and improve the outcome regarding the number of lymph node resections [14].

We expanded the application of 3D printing to locally advanced pancreatic ductal adenocarcinoma (PDAC) to optimize the novel therapy of magnetic hyperthermia with magnetic nanoparticles (MNPs). Tumor treatment with MNPs is an innovative approach that is already used in patients with tumors located at the surface of the body [15]. Bound within a thermosensitive layer, the MNPs and a chemotherapeutic agent are injected into a peripheral vein and then accumulated at the tumor site via a magnetic field trap consisting of a combination of permanent magnets and electromagnetic coils. After applying an alternating magnetic field, the thermosensitive layer melts and releases the drug locally. In addition, the tumor is treated with magnetic fluid hyperthermia at 41–44 °C [16]. The MNPs, which are already often used as contrast agents for magnetic resonance imaging, are secreted via the liver and the kidney. We have proved by establishing a biophysical model that the magnetic field traps can also be placed endoscopically to treat tumors inside the body [1]. Additionally, we showed that magnetic hyperthermia diminishes the clonogenic potential of human pancreatic adenocarcinoma cell lines by combining intracellular nanoheating and extracellular bulk heating [16]. Its effect on pancreatic cancer organoids was demonstrated as well [17]. Resection is the only curative treatment option for pancreatic ductal adenocarcinoma but often the tumor is already locally advanced at the time of diagnosis with the infiltration of the superior mesenteric artery (SMA) located behind the pancreas body. By applying magnetic hyperthermia in a neoadjuvant setting, secondary resectability may be achieved. In the case of PDAC, the magnetic field trap should, therefore, be placed on the posterior wall of the stomach as the pancreas and the SMA are directly adjacent to it. For applying this form of therapy in patients, it will be most practicable to place the magnetic field trap endoscopically and then fixate it at the correct position in the stomach to allow for the repetition of treatments without the patient having to undergo sedation with endoscopy every time. Hence, it is necessary to place the magnetic field trap in close proximity to the locally advanced pancreatic cancer and in a manner that it will not be displaced after a few days. An ideal magnetic field trap may be found via our biophysical model [1]. As the porcine anatomy clearly differs from the human anatomy, three-dimensional replicas of the patients’ organs are more suitable to represent the human anatomy, especially in an individualized treatment approach. Therefore, the ideal position for placing the magnetic field trap with respect to the main region of interest, the tumor site infiltrating the vessels, may be found using 3D replicas from patients’ digital data. 

We anticipate that 3D-printed replicas of patient organs offer new insights into the individual anatomy. The primary goal of our project was to show the feasibility of replacing animal experiments for pre-clinical cancer treatment optimization with experiments on 3D-printed replicas. A tumor treatment with magnetic nanoparticles served as an example. Our further goals were (a) to develop a widely available method to fabricate a 3D-printed replica from individual patient data and (b) to test mechanical aspects of the field trap attachment at the right place near the tumor. The fabrication method includes a morphologic box of experimental choices and is described thoroughly to allow users to customize the method and apply it in their own experiments. With this description and by use of solely open access software, our process can be learned by anyone, even inexperienced users, within a short time.

## 2. Materials and Methods

### 2.1. Development of Patient-Specific 3D Replicas 

We first focused on developing a transparent method to fabricate a patient-specific 3D replica, allowing for a broad and universal scope of application. Therefore, the interdisciplinary team of surgeons, endoscopists, mechanical engineers, and radiologists aimed to create a user-friendly protocol. It was important to us that even inexperienced students could be able to learn the steps of our method within a short time. Following Bucking et al. [18], we divided the process into three parts: image segmentation, mesh refinement, and 3D printing. As a validation of the developed method, we additively manufactured the pancreas and its surroundings of a patient with locally advanced PDAC with a Lulzbot Mini 3D printer (Fargo, ND, USA).

The procedure started with computer tomography (CT) data from a specific patient. The use of patient-related data was approved by the local institutional review board (IRB) (EK 030/19) and conducted in accordance with the Declaration of Helsinki. The CT data were processed and reproduced as 2D-image slices. By segmentation of the relevant structures (for the validation case of locally advanced PDAC: pancreas, tumor, arteries, veins, stomach), we created a 3D mesh from the 2D image slices. The 3D mesh was processed further by smoothing and adding modifications for the later experiment, such as providing an option to open the stomach to investigate the position of the magnetic field trap more closely. Finally, a computer-readable code for the 3D printer, known as G-code, was generated that describes how to create the 3D-printed parts and support structures. 

The following overview describes our process in detail, including the various software programs employed. These programs were partly selected from steps found in the literature [18,19]. We aimed to use open access software.

Image segmentation:

Step 1. Carry out computer tomography scans (CT scans) as NIFTI (neuroimaging informatics) files of patients with locally advanced PDAC.
  Mesh refinement:
Step 2. Create a mesh in “ITK Snap”, version 3.8, by Paul Yushkevich and Guido Gerig [20]:
  - Marking of relevant structures (organs, vessels, etc.) on each slide with different colors;
  - Segmenting relevant structures (this makes a 3D mesh from 2D layer data). We generated 6 different meshes, the stomach, pancreas, veins, arteries, tumor, and splint;
  - Exporting as stereolithography (STL) file.
Step 3. Mesh refinement with Meshmixer, version 3.5.474, by Autodesk:
  - Smoothing (to reduce the striations from 2D layers and anomalies from the segmentation step);
  - Joining (if any segments were not fully connected during the segmentation step);
  - Adding hooks or other structures for physical model (to join and hold the different parts of the replica together more easily).
Step 4. Additional processing using Cinema 4D, version R19.053, student by Maxon:
  - Further mesh editing (e.g., making the flange of the stomach to be able to open and close it);
  - Optional video making.
Step 5. Importing into Lulzbot Cura 3.6.20 (printer-specific):
  - Selecting the default printing profile for polylactic acid (PLA) with a layer height of 0.18 mm; selected for a sufficiently high surface quality. Smaller layer heights will result in higher surface quality components.
  - Printing temperature changed to 230 °C as per the PLA material requirement;
  - Option to change amount of infill (infill is part of the 3D-printed part and not to be removed) and support (support should be removed);
  - Slicing of model and generating computer-readable G-Code (G-Code is code that tells the 3D printer how to move and create the part; it is mostly a series of commands dictating movement in x-, y-, and z-direction, nozzle temperature, and nozzle feed).
  3D printing:
Step 6. 3D printing on Lulzbot Mini (some machine parameters include: nozzle diameter of 0.5 mm, printing temperature of 230 °C, wire diameter of 2.85 mm):
  - Material was Polylite PLA by Polymaker;
  - Different colors by changing the filament spools: each spool was the same material type from the same manufacturer so print settings remained the same;
Step 7. Post-processing of 3D-printed replica:
  - Removal of infill or support material (breaking it out with pliers or sharp knives);
  - Manual grinding for surface smoothing with a Dremel 4000 tool (using alumina grit sanding bands);
  - Drilling holes with drill press or Dremel 4000 tool at relevant positions (for endoscope or tissue fixturing).
Step 8. Assembly of replica parts:
  - Using zip ties or thread to hold the replica parts together;
  - Tissue sewing.

During our printing evaluation, we measured the electrical power used through a power meter (“WATTS UP Pro Power Analyzer”, Electronic Educational Devices, Denver, CO, USA) between the 3D printer and the wall power plug. The area under the active power profile over time provided the electrical energy. With these data and the knowledge of local electricity costs, we could calculate the electrical energy costs. Furthermore, we gained insights into the environmental impact of 3D printing, as the generation of electricity is a major contributor to environmental impacts.

During the development of the process, the principles of product development were applied [21], in particular, interviews with customers and a discussion of customer needs, the creation of a morphologic box, and the discussion of design improvements. For example, the design question of using infill vs. support for the additive manufacturing and which basic shape (triangle vs. square) should be used was evaluated by three different students.

### 2.2. Experimental Design for a Magnetic Field Trap

To test the procedure described above, we produced a 3D-printed replica of the pancreas and its surroundings from a patient with locally advanced PDAC. To demonstrate that the 3D-printed model is, indeed, a replica of the patient’s original anatomy, we performed a CT scan of the replica model and overlayed these data with the original CT data. With this replica, we performed experimental design investigations for a magnetic field trap. Hereby, we searched for the best position for the endoscopic placement of the magnetic field traps needed at the posterior stomach wall to achieve a high magnetic field at the region of interest (the tumor infiltrating the superior mesenteric artery). For measurement of the magnetic field, a 3-axis “Go-Direct GDX-3MG” sensor (Vernier, Beaverton, OR, USA) was used. In addition, the replica was used to practice the endoscopic view of the right position to be able to position the magnetic field trap in a patient more easily. Endoscopy on the 3D replica was realized by three different experienced endoscopists. 

As an optimized magnetic field trap needs to stay in place for a couple of days when employed in patients, we investigated methods for best fixation of the magnetic field trap at the stomach wall. As the easiest method, we added loops at both ends of the magnetic field trap and fixated them to the stomach wall with endoscopic clips (Olympus, Tokyo, Japan). We were also able to additionally train the endoscopists in the realistic endoscopic handling and the fixation by using porcine animal cadavers, acquired from other approved animal experiments directly after euthanization. No ethical approval was needed as we did not work with living animals according to German law and the guidelines of the ethics committee of the LANUV (State Agency for Nature, Environment and Consumer Protection, North Rhine-Westphalia, Germany). The magnetic field trap used was designed as previously described in [1] and coated with latex rubber (see Figure 12). After endoscopic placement and fixation of the magnetic field trap, we explanted the stomach and measured the forces that still held the magnetic field trap in place with a spring scale (“SI Manufacturing”, Ontario, Canada) as a model for the real-life situation.

## 3. Results

### 3.1. Development of Patient-Specific 3D Replica

**Figure 1:**
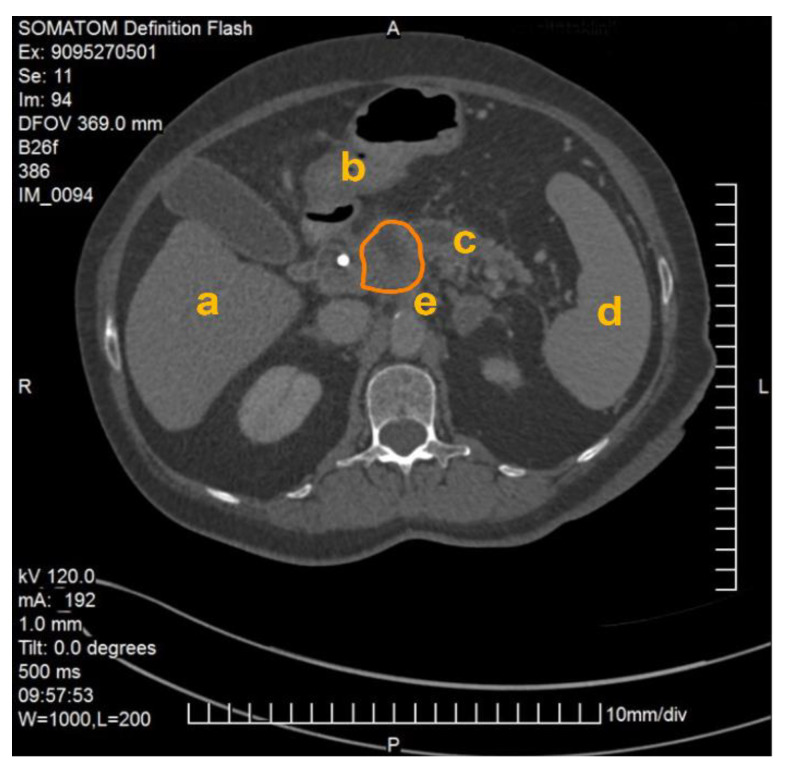
CT scan of a patient with locally advanced pancreatic ductal adenocarcinoma. Visible are the pancreas with a splint in the head of the pancreas as well as the liver (**a**), the gastroduodenal junction (**b**), the dilated pancreatic duct (**c**), the spleen (**d**), the abdominal aorta and the origin of the coeliac trunc (**e**). The PDAC tumor is outlined in orange.

The method described was applied and validated on a patient with locally advanced PDAC. Figure 1 demonstrates one slide of the total CT scan. As this selected patient reported with jaundice, a splint was placed in the common bile duct. The images were exported as NIFTI files and anonymized (Step 1) before being further processed in the program ITK Snap (Step 2).

**Figure 2:**
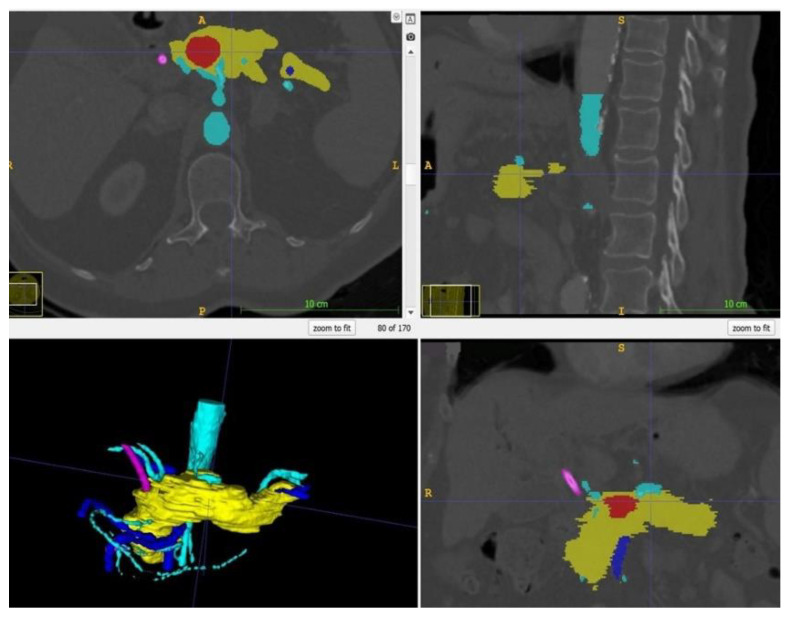
Screenshot of ITK Snap with the tumor in red, the pancreas in yellow, the arteries in light blue, the veins in dark blue and the splint in pink.

**Figure 3:**
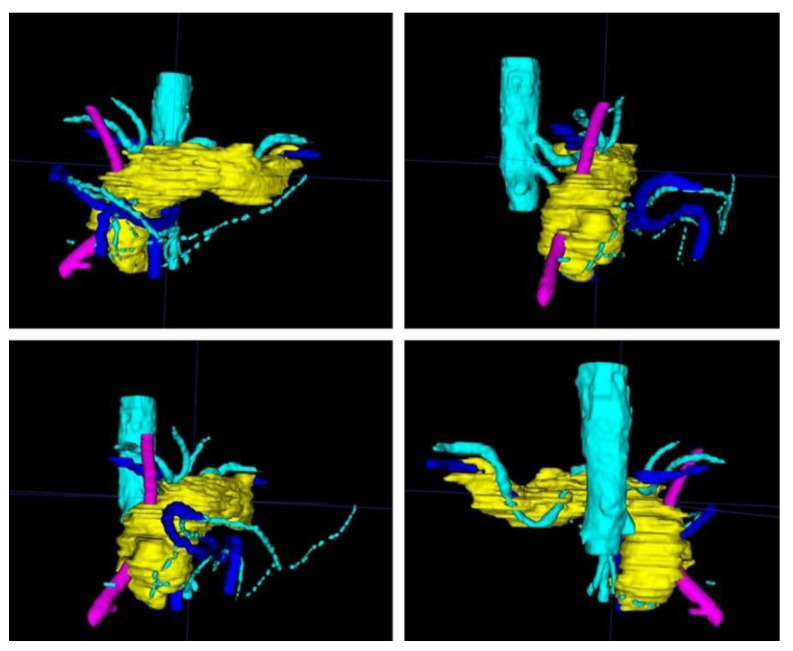
After completion of the final mesh, the pancreas and its surroundings can be viewed from different angles. Note the rather rough surface as the CT scans had a slice thickness of 1 mm. (The pancreas in yellow, the arteries in light blue, the veins in dark blue, and the splint in pink.)

Within ITK Snap, the relevant structures, i.e., in this case the stomach, the pancreas, the tumor and the arteries and veins, were marked on each slide with different colors. Then, a 3D mesh was made from the 2D layer data (Step 2.b). The different meshes were exported as STL files. Figure 2 displays a screenshot of ITK Snap and Figure 3 demonstrates views from different angles on the created meshes.

**Figure 4:**
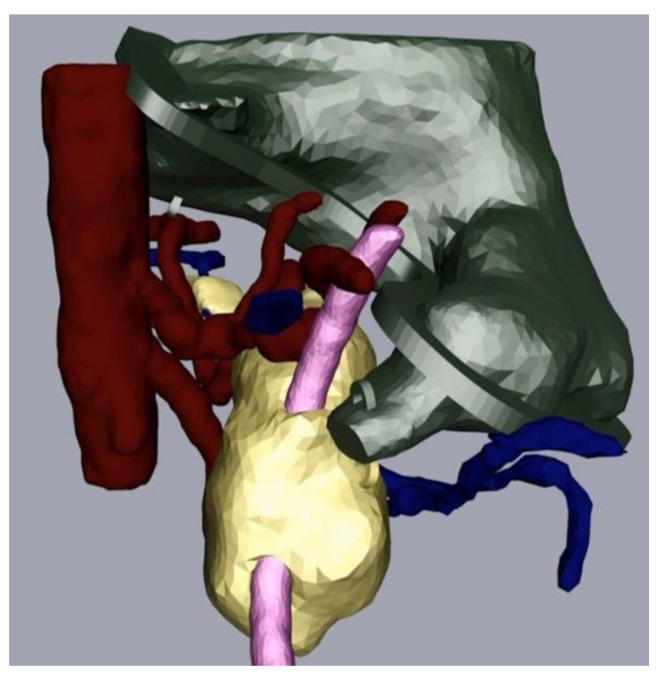
Pancreas (yellow), arteries (red) veins (blue), stomach with flange and hooks (green) and splint (pink) after processing.

The next part of the process was the mesh refinement as described above. For this, we smoothed the mesh with the program Meshmixer (Step 3a) and added hooks to be able to join the physical replica parts together with strings later. We also performed additional processing in Cinema 4D (Step 4). In our case, this consisted of adding a flange as we wanted to be able to open and close the stomach. We made a video that displayed the different parts of the replica coming together (available on request). Figure 4 depicts the assembly after processing and before the last level of 3D printing.

The meshes of the organs were then imported into the 3D printer-specific software Cura, for slicing and printing (Step 5). The infill (density) of the 3D-printed part and the support structure (removed after printing) had to be chosen. To find the most suitable method, several set-ups of infill and support with different bases (squares, triangles, etc.) were chosen. Then, a G-code file for printing was generated to dictate printer movements and filament feed for each print. STL models from the CT scans have a wall thickness of 0 by design as the structures are only marked by lines on each CT scan slide. In order to print them properly, the wall thickness had to be set to at least the minimum of the printing thickness (defined by the nozzle size). When creating cutting planes in the STL model such as the stomach halves, the cut plane will become a new surface. To avoid printing this “new” surface, the roof thickness was set to be 0 in Cura.

**Figure 5:**
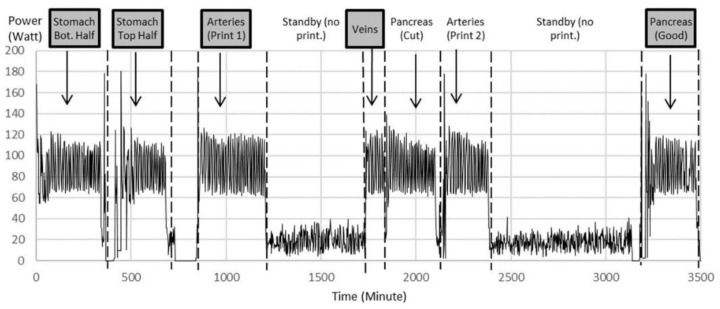
Power data during printing of multiple iterations of organ parts as examples to show power variation during printing between 120 W and 60 W with an average of 90 W. The printer consumes an average of 20 W during standby, when filament spools were exchanged and the corresponding printing code was uploaded to the printer. Notice multiple iterations for arteries and pancreas. Highlighted are the printed parts chosen for further experiments.

Organ replicas were then 3D printed on the Lulzbot Mini (Step 6). The standard quality with a layer height of 0.18 mm was proven to be sufficient. The power monitoring of the 3D printer as shown in Figure 5 displays the power for 3D printing of different organs and power for standby times over the whole printing procedure of nearly 6 h for an example printing process. Notice the multiple iterations for arteries and pancreas.

From this, the electrical energy could be calculated. In California, where this study was performed, 1 kWh of electrical energy costs about USD 0.13 at the time of this experiment. Table 1 shows the material and electricity costs for 3D printing, excluding the labor. The standby time and material costs for the support were negligible. The material costs (PLA filament) were USD 25 per kg.

**Figure 6:**
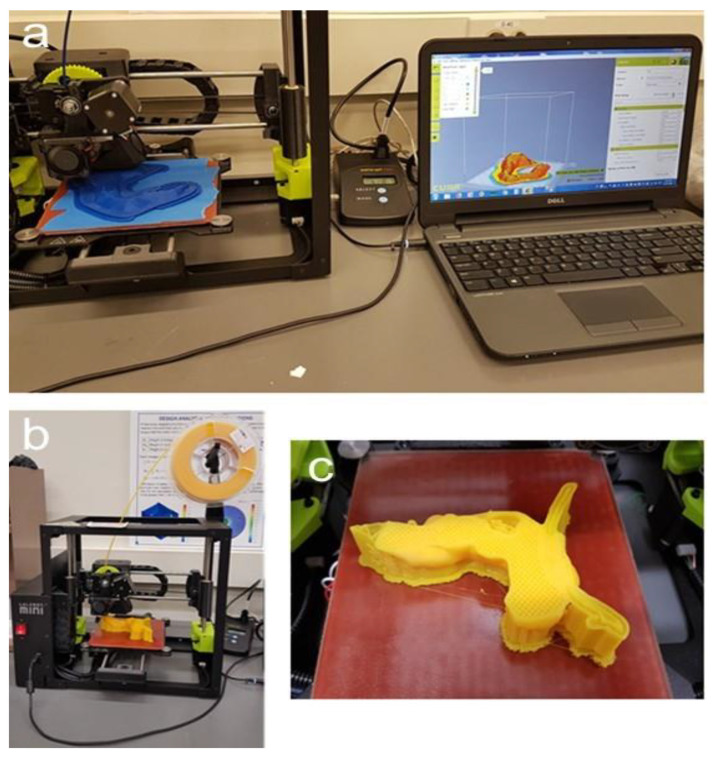
Three-dimensional printing of the replica. (**a**): Printing of the stomach on the Lulzbot Mini with the power meter in the middle and software, Cura, displayed on the computer; (**b**): Lulzbot Mini with filament spool; (**c**): 3D-printed pancreas during the process of printing with stable infill, the supports, the splint, and a cavity at the tumor site where the magnetic field can later be measured.

Figure 6 depicts the 3D-printing process. Notice that the pancreatic tumor itself was not printed, thereby leaving a cavity in order to measure the magnetic field strength at the tumor site in additional experiments.

**Figure 7:**
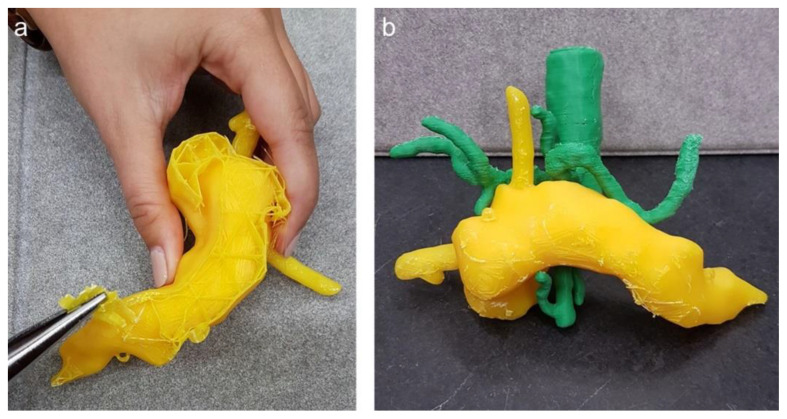
(**a**) Post-processing of the 3D pancreas replica with splint; (**b**) pancreas with aorta, coeliac truncus and superior mesenteric artery.

After successful 3D printing of the different organs, the post-processing of the replica was started (Step 7), which included the extraction of the support material as well as manual grinding for smoothness and drilling holes at relevant positions, i.e., at the cardia for the endoscope to enter the stomach. Figure 7 displays the manual extraction of the support material needed for 3D printing as well as an assembly of the pancreas with the vessels.

### 3.2. Further Design Improvements

Aiming to provide a broad scope of possible applications, common user needs were specified: Must requirements are compulsory, whereas can/optional requirements are beneficial to have, but can be skipped depending on time and availability. The following list gives examples for our application and can be adapted to other applications and is not necessarily all-encompassing.

1. Must/compulsory requirements for the 3D-printed replica:
  - Must mirror the anatomical characteristics of a patient as closely as possible (organ size, shape and location);
  - Must use the stomach, proximal end of duodenum, pancreas, and relevant vessels;
  - Stomach must be able to attach to the porcine stomach wall;
  - Single 3D-printed organs have to fit together;
  - Must hold the possibility to measure a magnetic field at the tumor site.
2. Can/optional requirements for the 3D-printed replica:
  - Should mirror the haptic characteristics of the organs;
  - Organ shapes can be personalized to different patients (satisfied in our application);
  - Can mirror the elasticity of organs;
  - Can mimic the mechanical characteristics of the tissue;
  - Can open the anterior wall, which can be used to look inside (satisfied in our application);
  - Can use different colors for different organs (satisfied in our application);
  - Vessels can be hollow;
  - Bile duct can be hollow, which could be used to help the operating room strategy.

**Figure 8:**
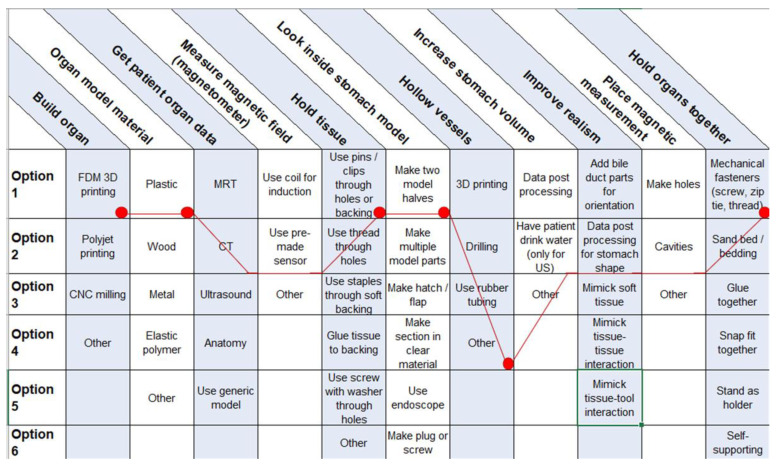
Morphologic box for 3D organ replicas with special regard to endoscopic tumor therapy with MNPs. Red dots represent our choices.

To guide the users in their decisions, a morphologic box was created (Figure 8). The functions in the first column are met by selecting one of multiple options. Users can choose between these options and create a unique solution.

### 3.3. Experimental Results

**Figure 9:**
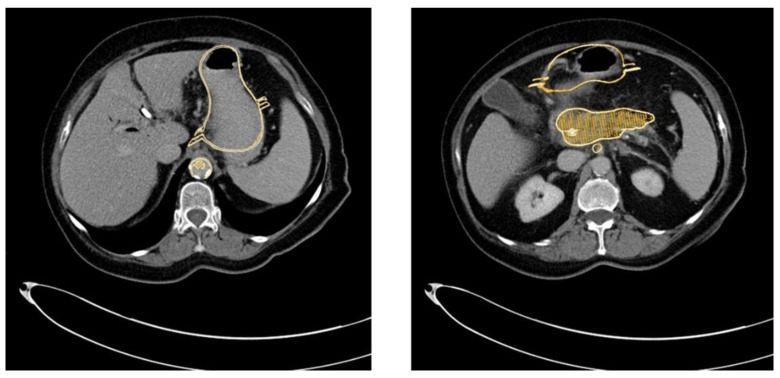
Overlay of the CT scan and the 3D-printed model (golden) and the original patient’s CT scan, demonstrating the high congruency between the replica and the original data.

Our 3D-printed replica of the pancreatic tumor and its surroundings mirrored the real-life situation in patients very closely, as evaluated by three different endoscopists. The CT scan of the 3D-printed replica proved the high congruency between the model and the original patients’ data (Figure 9).

**Figure 10:**
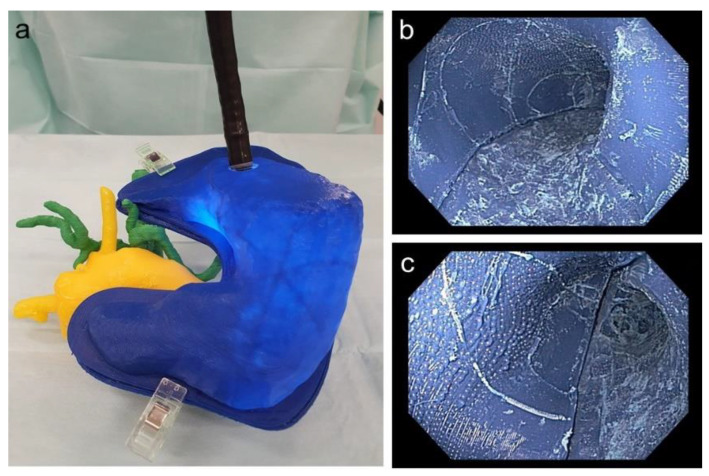
(**a**) Endoscope inside the stomach with adjacent printed pancreas as well as vessels; (**b**) endoscopic view of the corpus of the printed stomach; (**c**) endoscopic view of the antrum of the printed stomach.

It was possible to perform a real-life comparable endoscopy of the stomach and practice the application of a magnetic field trap. Figure 10 depicts the endoscope to place the magnetic field trap inside the 3D replica as well as an endoscopic view inside the 3D-printed stomach.

**Figure 11:**
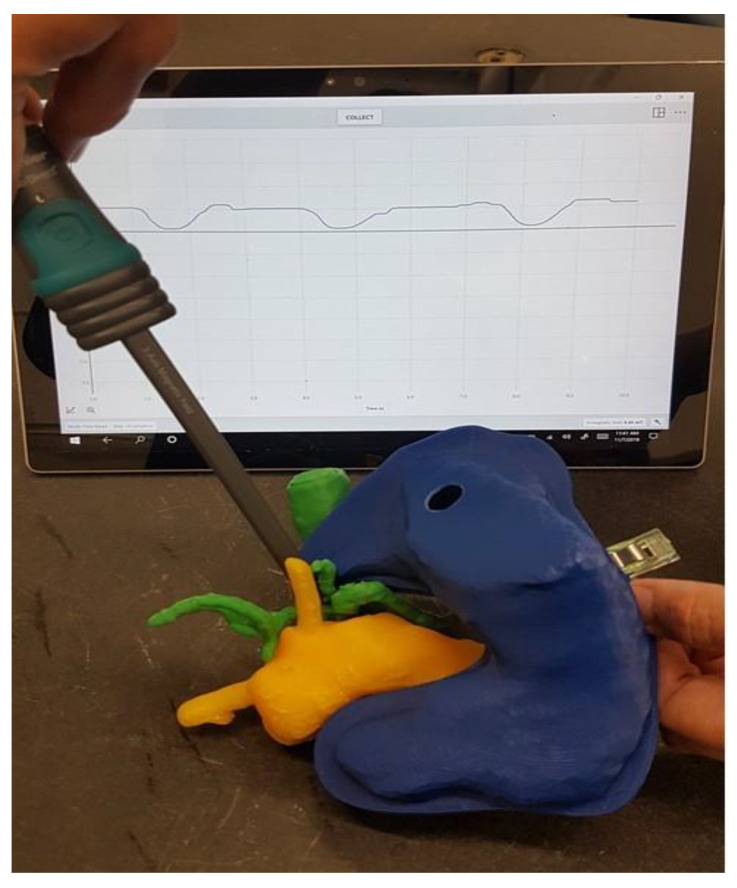
Measurement of the magnetic field where the tumor infiltrated the vessels.

The replica could then be used to determine the magnetic field at the tumor site dependent on the location of the magnetic field trap, taking into account the anatomy of the specific patient. Figure 11 shows the measurement of the magnetic field at the point where the tumor infiltrates the vessels in our 3D-printed replica with a magnetic field trap inside.

**Figure 12:**
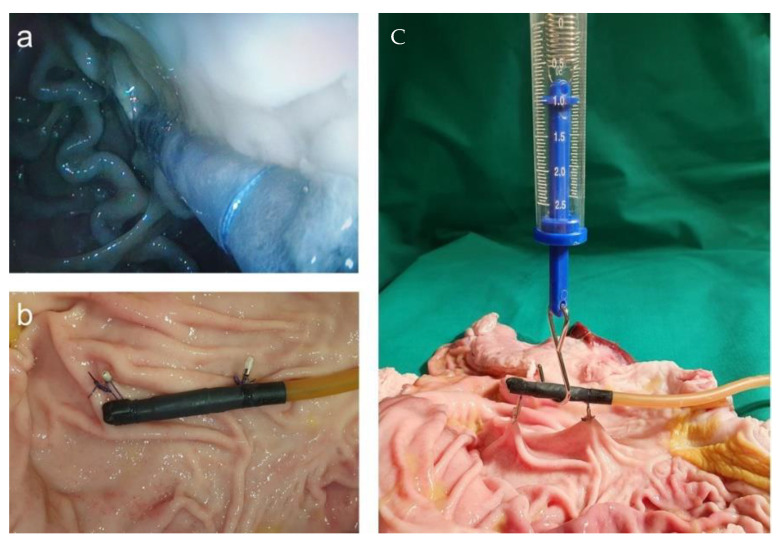
(**a**) Endoscopic view after the placement of magnetic field trap fixated with loops and endoscopic clips to the abdominal wall; (**b**) fixed magnetic field trap at the porcine wall; (**c**) strength measurements.

For handling the placement of the magnetic field trap in a position that could last for a couple of days, we used a porcine stomach. The simplest way to fixate the magnetic field trap was by adding loops and fixing them onto the stomach wall with endoscopic single-use clips. After opening the stomach, we performed strength tests with a force meter and found that the construction was stable up to at least 1 N. The endoscopic view after placement and the measurement of strength are displayed in Figure 12.

## 4. Discussion

With the described method, we were able to validate the printing of a realistic 3D replica of locally advanced human pancreatic cancer with the tumor in the pancreas together with the surrounding organs. The stomach was designed in two halves to allow it to be opened and to place magnetic field traps accurately. This model may be used for a variety of cancer-related research questions. We described the process of printing a 3D replica from CT scans in detail so that untrained students are able to adopt the method after a short training period. For the greatest level of accessibility, only open-source software was used throughout the process. This is the first time that the whole process of printing a 3D replica with open-source programs from CT scans has been described in detail. The morphologic box leads to a very versatile applicability to lots of cancer research questions.

The presented method also allows the use of porcine stomach from other animal experiments in line with the 3R (replace, reduce, refine) concept of animal welfare. This can either be achieved by incorporating the stomach in the 3D replica or as additional experiments on an explanted stomach. We found and validated a method to hold the magnetic field traps on the porcine mucosa in place efficiently for tumor treatment by the magnetic targeting of nanoparticles. Hence, we proved the feasibility of replacing animal experiments for pre-clinical optimization with experiments on 3D-printed replica, which was our primary goal. 

Using a power meter, we were able to measure the electric power consumed and calculate the electricity costs of the 3D-printing process. This is an extra aspect for the medical field to improve the research with regard to sustainability [22,23]. In addition, we calculated the material costs of the PLA filaments used. The calculation showed that the added costs of both material and electricity are quite low, being less than USD 4 per printed replica. Apart from the higher accuracy and the moral aspect regarding the “3R” principle, the costs for performing the experiments on a pig would be at least USD 900 for the animal and the narcosis.

Each organ has to be marked on each slice of the CT scan manually. Hence, the preparation of CT scans is time-consuming. Future research needs to address the potential benefit of artificial intelligence for this part [24,25,26,27]. Once the G-code from CT data is deduced, the replica can easily be re-printed in a larger quantity, thereby opening the possibility for extended teaching and training purposes [28,29]. 

During our work, we found several possibilities for the improvement of the 3D-printing settings, such as wall thickness and layer height, which we applied. In particular, for the 3D-printed replicas, an infill or support is needed because otherwise the parts will collapse during 3D printing. Infill is for hollow parts whereas support structures are needed for overhanging parts. Most printers will have different settings for infill or support structures. It is advised to choose a model orientation on the printer bed, so that mainly supports instead of infill are generated. Supports are designed to be removed easily, whereas infill is more demanding to take away. If not avoidable, triangle infill is more easily removed than other infill structures. A lower infill percentage also allows for easier removal but may result in a larger dimensional inaccuracy of the part. 

The interdisciplinary work of endoscopists, surgeons and radiologists on the one hand, and the mechanical engineers and physicists on the other hand, not only made this work possible, but also broadened the scope beyond the specific research question. By use of open-source software, the detailed description of the work steps, and including different requirements in the morphologic box, it is highly applicable to other research questions. This is in line with our subsidiary goal of developing a transferable method for 3D-printed replicas from individual patient data, which can easily be learned by inexperienced students within a short time. Three endoscopists performed a gastroscopy with the model and found it feasible. Nonetheless, this experience could be improved further as the internal surface of the prototype was only manually and rudimentarily processed. Further investigations need to address this aspect by the use of abrasive or chemical machining, as previously discussed for additively manufactured metal implants [30].

The replica was applied to find the best location of the magnetic field trap in a personalized treatment approach of tumor therapy with magnetic nanoparticles [1,16]. The replica provides the possibility of qualitatively testing the optimal position of the magnetic field trap to focus the energy on the desired spot. The ability to open the 3D-printed stomach makes it possible to examine the exact magnetic field trap position. In addition, porcine tissue may be attached inside the replica, if needed. 

With our 3D replica as well as with the explanted porcine stomach wall, we were able to find a stable fixation method to keep the magnetic field trap in place and attached to the interior stomach wall. This is an innovative method as treatments with magnetic hyperthermia after magnetic targeting have only been carried out for tumors on the surface of the body, so far [31,32]. We were the first to describe the feasibility of the endoscopic placing of magnetic field traps before [1] and provide here a method to fixate the coil formation in situ. The fixation with the application of loops and endoscopic clips holds the advantage that the patient has to undergo endoscopy and sedation only once. Thereafter, the targeting of the MNPs after injection into a peripheral vein can be performed several times by the electrical activation of the magnetic field trap. Due to the fixation with loops and endoclips, there will be shear forces that the fixation has to overcome. We were able to show that forces greater than 1 N are needed to dislocate the magnetic field trap. It is known that endoscopic clips, e.g., to stop a gastrointestinal bleeding, remain in place for at least 1–3 weeks [33,34]. Nonetheless, further experiments are required to investigate how long the magnetic field trap will stay in place in vivo. Still, with the results from the described experiments, fewer animal experiments will be needed. 

The limitations of our study are that we only used PLA filament for printing, which does not reflect the elasticity of the human tissue correctly. Further experiments with other 3D-printing materials will be able to adjust to more realistic conditions but concerns regarding safe application when additives are printed with the PLA filaments have to be taken into consideration [35]. Additionally, it is difficult to print very fine vessels. Hence, the arteries had to be printed twice due to printing errors in the first print. Still, we included theses data in our cost analysis to be able to calculate the real costs. As patients usually fast before a CT scan and their stomachs are empty, the user has to be aware that the CT data are different from endoscopic conditions, where the stomach is generally blown up with air. Nonetheless, for the particular application of tumor treatment with MNPs, the user has to carefully consider the flexible size of the stomach needed for the replica. The fully blown-up stomach is the situation during endoscopy on the one hand, as opposed to the empty stomach, which is the situation during tumor treatment with endoscopically placed nanoparticles on the other hand. Through the standardization of the conditions between image acquisition and intervention, it should, however, be possible to generate a high degree of consistency through the application of defined amounts of fluid to the stomach and constant patient positioning. Another limitation is that the magnetic field values needed for the endoscopic targeting of MNPs in the case of locally advanced PDAC are not described in the literature yet. Nonetheless, the measured magnetic field values are consistent with a proof-of-principle study performed by us (unpublished data). As the model is non-dynamic and cannot take aspects such as magnetic particle penetration within the tissue into account, further animal studies are needed, but the number of animals necessary may be reduced to a large extent in line with the “3R” rules. 

## 5. Conclusions

Our study demonstrates that the development of 3D replicas from clinical CT data is possible and may be used for preclinical studies. The detailed description of the workflow and use of open access software allows producing 3D replicas for research or training purposes, even by inexperienced people, within a short time. The 3D replica gives insights into the human anatomy more precisely than standard animal experiments. In addition, the model can be easily produced at low cost in high quantities, for example for teaching or research. With this, the number of animal experiments can be reduced, the repetition accuracy may be maximized and different experiments are possible on the same model.

## Figures and Tables

**Table 1:** Material and electricity costs for the finished printed replica.

Replica	Material Costs	Electricity Costs
Pancreas	52.56 g × 0.025USD/g = USD 1.314	350 min × 90 W × 0.13 USD/kWh = USD 0.068
Stomach	57.78 g × 0.025USD/g = USD 1.445	600 min × 90 W × 0.13 USD/kWh = USD 0.117
Vessels	17.12 g × 0.025USD/g = USD 0.43	400 min × 90 W × 0.13 $/kWh = USD 0.078
Total costs	USD 3.189	USD 0.263

## Data Availability

The data presented in this study are available on request from the corresponding author.

## Abbreviations

3D	three dimensional
3R	reducing, refining and replacing animal experiments
CO	Colorado,
CT	computer tomography
G-code	computer readable code for the 3D printer
IRB	institutional review board
kWh	kilo Watt hour
LANUV	State Agency for Nature, Environment and Consumer Protection, North Rhine-Westphalia
MNP	magnetic nanoparticles
N	Newton
NIFTI	Neuroimaging Informatics
PDAC	pancreatic ductal adenocarcinoma
PLA	polylactide acid
SMA	superior mesenteric artery
SLS	selective laser sintering
STL	stereolithography
USA	United States of America
USD	US Dollar
W	Watt
